# The Regeneration of the Body

**Published:** 1890-06

**Authors:** 


### THE REGENERATION OF THE BODY.

The soul can be regenerated and the body remain disorderly ; the body can be trained to a fine physical life and action and the soul remain unregenerate ; but certainly the *fulness of life*, both for this world and the next, must come from a more perfect harmony of the material body with the soul.

So long as the soul needs the body at all, it must be of inestimable importance that the body should conform itself to the pure laws of nature by shunning physical evils, as it is that the soul should be born again through shunning spiritual evils. The life of both comes from looking to the Lord.—*Kindergarten.*

